# Estimation Model for Maize Multi-Components Based on Hyperspectral Data

**DOI:** 10.3390/s24186111

**Published:** 2024-09-21

**Authors:** Hang Xue, Xiping Xu, Xiang Meng

**Affiliations:** 1 College of Electronic and Information Engineering, Beihua University, Jilin 132021, China; xuehang@beihua.edu.cn; 2 College of Optoelectronic Engineering, Changchun University of Science and Technology, Changchun 130022, China; xxp@cust.edu.cn

**Keywords:** hyperspectral imaging, maize, quality detection, non-destructive, machine learning

## Abstract

Assessing the quality of corn seeds necessitates evaluating their water, fat, protein, and starch content. This study integrates hyperspectral imaging technology with chemometric analysis techniques to achieve non-invasive and rapid detection of multiple key components in corn seeds. Hyperspectral images of the embryo surface of maize seeds were collected within the wavelength range of 1100~2498 nm. Subsequently, image segmentation techniques were applied to extract the germ structure of the corn seeds as the region of interest. Seven spectral data preprocessing algorithms were employed, and the Detrending Transformation (DT) algorithm was identified as the optimal preprocessing method through comparative analysis using the Partial Least Squares Regression (PLSR) model. To reduce spectral redundancy and streamline the prediction model, three algorithms were employed for characteristic wavelength extraction: Successive Projections Algorithm (SPA), Competitive Adaptive Reweighted Sampling (CARS), and Uninformative Variable Elimination (UVE). Using the original spectra and extracted characteristic wavelengths, PLSR, BP, RBF, and LSSVM models were constructed to detect the content of four components. The analysis indicated that the CARS-LSSVM algorithm had the best prediction performance. The PSO algorithm was employed to further optimize the parameters of the LSSVM model, thereby improving the model’s prediction performance. The R values for the four components in the test set were 0.9884, 0.9490, 0.9864, and 0.9687, respectively. This indicates that hyperspectral technology combined with the DT-CARS-PSO-LSSVM algorithm can effectively detect the main component content of corn seeds. This study not only provides a scientific basis for the evaluation of corn seed quality but also opens up new avenues for the development of non-destructive testing technology in related fields.

## 1. Introduction

As the third-largest grain crop in the world, maize not only plays a significant role in the human diet, but also serves as an important raw material for industrial production. The quality of its seeds is directly related to the yield of corn and the quality of its derivative products. The accurate and efficient detection of component content in corn seeds is of great significance in elevating the overall standards of the corn planting industry and promoting the growth of related industries. Among the various components of corn seeds, the water, fat, protein, and starch content are key indicators for evaluating seed quality. Water content is an important factor affecting seed storage stability, transportation safety, and germination rate after sowing. High levels of moisture content can accelerate seed respiration, consume nutrients, and even lead to mold and decay; however, too low a water content may cause the seeds to lose vitality and affect germination. Fat, protein, and starch are the main components that constitute the nutritional and economic value of corn seeds, and their content directly determines the nutritional quality and market value of corn. Conventional methods for detecting the composition of corn seeds, such as drying and titration, can meet detection needs to some extent, but they have the disadvantages of being time-consuming, cumbersome to operate, and causing damage to samples, making it difficult to fall short of meeting the needs of rapid, non-destructive, and batch detection in modern agricultural production. Hence, investigating a novel and effective detection technology holds immense importance in enhancing the efficiency and precision of determining the compositional content of corn seeds.

Hyperspectral imaging technology (HSI), an advanced detection method that amalgamates traditional near-infrared spectroscopy with machine vision technology, has gained significant traction in the realm of agricultural product quality assessment in recent years. This technology facilitates swift, non-invasive, and precise analysis of sample composition by capturing spectral data from every pixel on the sample surface and integrating it with spatial information. Over the past few years, it has been subject to extensive research and application in the quality evaluation of agricultural products and food items. [1,2,3,4,5]. Liu et al. established an SSA-SVM model based on hyperspectral data to predict the protein content of milk. The prediction results met the accuracy requirements for dairy testing, providing a feasible new method for the rapid detection of milk protein content [6]. Xuan et al. established a multivariate linear regression model for protein content in rice grains based on HSI, with an $R_{c}^{2}$ of 0.9393 in the validation set, providing the possibility for non-destructive protein content detection [7]. Van Haute established a quantitative analysis model between carotene and spectrum based on LSSVM and PLSR, accurately predicting the content of carotene in carrots [8]. Khajehyar et al. used hyperspectral signatures to predict foliar nitrogen and calcium content of tissue-cultured little-leaf mockorange (Philadelphus microphyllus A. Gray) shoots, and the $R^{2}$ values of the established RF regression model reached 0.72 and 0.99 [9]. Zhang et al. combined hyperspectral technology with deep learning algorithms to establish a CNN-LSTM corn seed moisture content detection model, achieving a prediction set *R*^2^ of 0.937 [10].

In summary, hyperspectral imaging has great potential in predicting crop component content. However, previous studies were often limited to specific producing areas and focused on the detection of a single component in crops. To comprehensively enhance the breadth and depth of crop component detection, this study took corn samples from different provinces in China as the subjects of study, ensuring the diversity and representativeness of the data. On this basis, by integrating HSI with advanced data processing and analysis methods, an HSI-based multi-component content detection model for corn seeds was established, offering a scientific foundation and technical support for quality control and production management in the corn seed industry.

## 2. Materials and Methods

### 2.1. Samples

**Figure 1:**
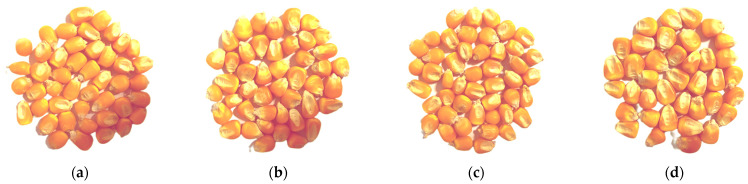
Images of several different types of corn seeds. (**a**) XH825, produced in Jilin Province, China; (**b**) YS679, produced in Gansu Province, China; (**c**) JY205, produced in Henan Province, China; (**d**) HD136, produced in Hainan Province, China.

The corn seed samples used in the experiment were provided by Jilin Guangde Agricultural Technology Co., Ltd. (Jilin, China). There were 80 varieties, all harvested in 2023, originating from Jilin Province, Hainan Province, Henan Province, and Gansu Province in China. These seeds vary in genetic background and growth environment, resulting in differences in the content of key components. For example, maize grown under drought conditions may contain lower water and higher starch content to cope with water stress. The seeds selected as the research objects are aimed at covering a wide range of genetic backgrounds and ecological adaptability, ensuring the broad representativeness and universality of the research results. Seeds with plump grains and no defects were chosen from each variety for testing. Figure 1 shows photos of seeds from four of them.

To achieve a stable condition of the seeds and ensure consistency in experimental conditions, prior to the experiment, all seeds were left to stand in the same experimental environment for more than 72 h, followed by the collection of hyperspectral images. Subsequently, each variety was divided into four aliquots, and their moisture, fat, protein, and starch contents were determined using chemical quantitative methods. Finally, a dataset correlating spectral data with the contents of these components was established.

### 2.2. Experimental Equipment

**Figure 2:**
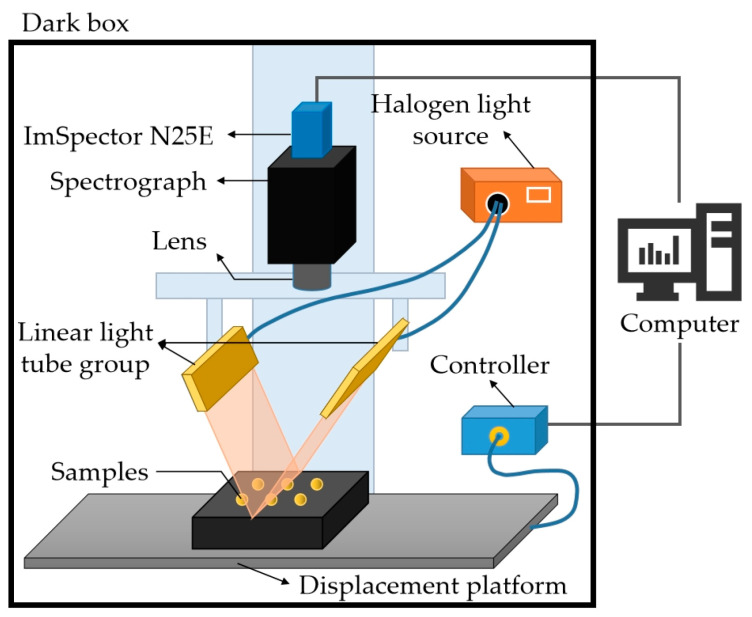
Structure of hyperspectral image acquisition system.

The experiment utilized a push-broom visible-to-near-infrared hyperspectral imaging system to collect spectral data from corn samples. The system comprises a 1000–2500 nm imaging spectrometer (ImSpertor N25E, Spectral Imaging Ltd., Oulu, Finland); a 1600 × 1200 pixel CCD camera (Bobcat 1410, IMPERX Inc., Boca Raton, FL, USA); a bilateral 150 W halogen light source (IT3900, Illumination Technologies Inc., Liverpool, NY, USA); and a one-dimensional precision mobile platform (IRCP-0076-400, Isuzu Optics Corp., Taiwan, China). During image acquisition, the entire system is placed in a closed dark box to prevent interference from ambient light. The structure of the acquisition system is shown in Figure 2. The scanning mode of the system is line scanning, with a spectral acquisition range of 935.5–2539 nm and a resolution of 6.3 nm. In the experiment, through repeated debugging, the lens height was set to 44 cm, the exposure time was set to 10 ms, and the speed of the platform movement was set to 8 mm/s, allowing for the collection of clear images of corn kernels.

Due to the optical properties of the hyperspectral imager’s lens, it generates a large amount of dark current, and the distribution of light intensity also exhibits non-uniformity in certain specific wavelength bands. These factors can result in images obtained from the hyperspectral imager being riddled with a lot of noise. Therefore, to extract useful information from hyperspectral images, it is necessary to perform correction processing on the original image to improve its quality. The image correction formula can be expressed as:

(1) $$R=\frac{I_{o}-I_{dark}}{I_{white}-I_{dark}}$$

where *R* represents the corrected image, *I_o_* denotes the original sample image, *I_white_* signifies the image captured by scanning the standard whiteboard, and *I_dark_* represents a dark field image obtained by covering the camera lens during the experiment

### 2.3. Method and Principle

#### 2.3.1. Measurement of Physical and Chemical Indicators of Corn Seeds

##### Method for Determining Moisture Content

According to the crop seed inspection procedures GB/T3543.6-1995 [11], the direct drying technique is used to determine the moisture content inside the seeds. The specific steps are as follows: first, grind the seed sample thoroughly to make the individual particle size less than 2 mm, then weigh the sample, select 10 g of the sample and gradually add it to the weighing bottle, then dry it in a drying oven for 4 h, and finally cool the ground seed particles at room temperature. After cooling for 0.5 h, weigh them again. Repeat the above procedure, remove and cool the seeds after each hour of drying, and then weigh them until the weight is stable. The moisture content in the seeds is:

(2) $$C_{moisture}=\frac{M_{2}-M_{3}}{M_{2}-M_{1}}\times 100\%$$

where $M_{1}$ represents the weight of the sample tray (g); $M_{2}$ denotes the weight of the sample tray and the sample before drying (g); and $M_{3}$ represents the weight of the sample tray and the sample after drying (g).

Each sample is measured three times, and the average value is taken as the moisture content of that sample. This results in 80 sets of hyperspectral data and corresponding moisture content values, which can be used as the dataset for the moisture content prediction model.

##### Method for Determining Fat Content

The fat content in corn samples was measured using the Soxhlet extraction method as specified in the national standard GB5009.6-2016 [12]. This method employs the principles of solvent reflux and siphonage to ensure that solid substances are completely extracted by pure extraction agents in each operation, significantly enhancing extraction efficiency. In the experiment, petroleum ether was used as the solvent, due to the difference in boiling points between the fat and the solvent in the sample. Therefore, the solvent can be removed by heating and evaporation. After several solvent refluxes, the petroleum ether is completely evaporated, leaving only the sample fat content. Finally, the true value of the fat content can be calculated based on the difference in mass before and after drying the sample. The formula for calculating the fat content ratio is as follows:

(3) $$C_{fat}=\frac{M_{1}-M_{0}}{M_{2}}\times 100\%$$

where $M_{1}$ represents the total weight of the flask and fat (g); $M_{0}$ denotes the mass of the flask (g); and $M_{2}$ signifies the mass of the sample (g).

##### Method for Determining Protein Content

The protein content of corn kernels is determined using the Kjeldahl nitrogen determination method, in accordance with GB5009.5-2016. The underlying principle is that protein is decomposed under catalytic heating conditions, and the resulting ammonia combines with sulfuric acid to form ammonium sulfate. Alkaline distillation releases the ammonia, which is absorbed by boric acid and then titrated with sulfuric acid or hydrochloric acid standard titration solution. The nitrogen content is calculated based on the amount of acid consumed, and then multiplied by a conversion factor to obtain the protein content. The formula for calculating the protein content is as follows:

(4) $$C_{protein}=\frac{\left(V_{1}-V_{2}\right)\times c\times 0.014}{m\times V_{3}/100}\times F\times 100$$

where $V_{1}$ is the volume of standard titrant of sulfuric acid or hydrochloric acid consumed by the test solution (mL); $V_{2}$ is the volume of standard titrant of sulfuric acid or hydrochloric acid consumed by the reagent blank (mL); c is the concentration of sulfuric acid or hydrochloric acid standard titrant (mol/L); 0.014 refers to the nitrogen content (g) equivalent to 1.0 mL of sulfuric acid or hydrochloric acid standard titration solution; m is the mass of the sample (g); $V_{3}$ is the volume of the aspirated digestive juice (mL); and F is the coefficient for nitrogen conversion to protein, which is taken as 6.25 here.

##### Method for Determining Starch Content

The starch content of corn kernels is determined by enzymatic hydrolysis, with reference to GB5009.9-2016. The main process involves the following steps: after removing fat and soluble sugars from the sample, the starch is hydrolyzed into disaccharides using amylase, and then hydrolyzed into monosaccharides using hydrochloric acid. Finally, it is measured as reducing sugars and then converted into starch. The formula for determining the starch content is:

(5) $$C_{starch}=\frac{\left(A_{1}-A_{2}\right)\times 0.9}{m_{1}\times 50/250\times V_{1}/100\times 1000}\times 100$$

where $A_{1}$ represents the content of reducing sugar (mg) in the sample for determination; $A_{2}$ denotes the content of reducing sugar (mg) in the reagent blank; 0.9 is the conversion factor for converting reducing sugar (calculated as glucose) into starch; $m_{1}$ represents the mass of the weighed sample (g); and $V_{1}$ indicates the volume of the sample processing solution used for measurement (mL).

#### 2.3.2. Preprocessing Methods

The spectral signal of complex samples is frequently impacted by various factors, including noise, stray light, and baseline shift, with the particle size of the sample being a pivotal factor in spectral measurement [13,14]. Specifically, with the increase in sample particle size, the reproducibility of the spectrum decreases, while the variability of the spectrum increases exponentially. Due to the variations in shape and diameter of individual corn kernels, the resulting spectral data show significant differences, which further complicates the elimination of measurement errors and ultimately impacts the accuracy of quantitative analysis [15]. Therefore, to effectively mitigate spectral noise interference due to factors like seed morphology, it is crucial to employ suitable spectral preprocessing techniques to enhance the quality of the spectral data.

To enhance the quality of the spectral data and minimize noise and interference, seven preprocessing methods are applied to the spectral data: moving average (MA), Savitzky–Golay derivative convolution (SG), normalization (NOR), baseline correction (BC), multiplicative scatter correction (MSC), standard normal variate transformation (SNV), and detrending transformation (DT). Among them, the window size for MA is 7, the window size for SG is 7, the polynomial order for SG is 2, and the polynomial order for DT is 2.

#### 2.3.3. Feature Wavelength Extraction Algorithm

##### Successive Projections Algorithms (SPA) Method

SPA is a forward-selection technique that has been traditionally utilized for selecting feature wavelength variables, with the purpose of identifying the combination that reduces redundant information to the greatest extent [16]. The algorithm works by projecting each wavelength onto other bands and comparing the magnitudes of the projection vectors. It selects characteristic wavelengths with minimal redundant information and collinearity, ultimately reducing computation time and enhancing model classification accuracy. SPA selects only a small subset of wavelength variables from the original spectral data, yet these variables capture most of the useful information from the original data. This approach avoids information redundancy, significantly reduces model complexity, and improves the accuracy, speed, and robustness of predictions [17].

##### Uninformative Variable Elimination (UVE) Method

UVE is an algorithm for wavelength selection that utilizes PLSR coefficients. Its primary objective is to refine the modeling dataset by eliminating wavelength variables whose stability is poorer than noise, ultimately improving the predictive power of the model [18]. The method involves combining artificial random noise information with PLS to build a regression cross-validation model. By computing the ratio of the mean to the standard deviation of the regression coefficients, an evaluation metric is established to assess the importance of the characteristic wavelength variables. Furthermore, the maximum value of the noise matrix serves as the upper and lower limits for the algorithm’s threshold, with feature variables exceeding or falling below these thresholds being identified as the final optimized feature vectors.

##### Competitive Adaptive Reweighted Sampling (CARS) Method

The CARS feature extraction algorithm combines Monte Carlo sampling with PLS model regression coefficients [19]. By mimicking Darwin’s “survival of the fittest” principle, it uses adaptive re-weighted sampling (ARS) to progressively discard points with regression coefficients of lower absolute weights, thereby forming a new subset and re-establishing the PLS model. After several iterations, it selects the wavelength subset with the minimum root mean square error of cross-validation (RMSECV) from the PLS model as the characteristic wavelengths. This approach, widely used in spectral data analysis and image processing, effectively reduces data dimensionality and enhances model prediction accuracy.

#### 2.3.4. Modeling Algorithm

##### Partial Least Squares Regression (PLSR)

PLSR, a multivariate data analysis technique in statistics and machine learning, is particularly adept at addressing scenarios involving multicollinearity between dependent and independent variables [20,21]. It merges the strengths of principal component analysis (PCA) and multiple linear regression (MLR), with its central concept revolving around identifying novel orthogonal projection directions, or principal components, that optimize the covariance between the projected dependent and independent variables. This approach facilitates the establishment of a predictive model. Specifically, PLSR introduces latent variables by concurrently analyzing the relationship between the independent variable X and the dependent variable Y, and employs this to construct a linear model between them. Thanks to its unique principles and processes, PLSR is especially proficient in tackling high-dimensional data and small sample regression challenges [22,23].

##### Back Propagation Neural Network (BPNN)

The BPNN, a widely utilized multilayer feedforward neural network in supervised learning tasks, is trained through the error backpropagation algorithm. This model possesses robust nonlinear fitting capabilities, enabling it to automatically learn intricate relationships between input features [24]. A notable advantage of the BPNN model is its capacity to establish a nonlinear model using a limited set of refined wavelength variables. Structurally, it comprises an input layer, a hidden layer, and an output layer [25]. During the network’s learning process, the signal progresses through both forward and backward propagation. Specifically, the input sample is subjected to forward propagation, where it is sequentially processed by the hidden layer, starting from the input layer, and ultimately transmitted to the output layer. In cases where the actual output of the output layer diverges from the desired output, the error undergoes backpropagation. Subsequently, the weights and thresholds of each layer are adjusted along the gradient direction, progressively diminishing the error and ultimately achieving the target accuracy [26].

The number of hidden layer nodes can be determined by an empirical formula, which is calculated as follows:

(6) $$N=\sqrt{L+M}+\alpha$$

where *L* and *M* denote the node counts for the input and output layers, respectively, and *α* is an integer within the range of 0 to 10.

##### Radial Basis Function Neural Network (RBFNN)

RBFNN is an artificial neural network with a specific architecture, named for the radial basis function (RBF) used as the activation function in the hidden layer [27]. It has found widespread applications in fields such as function approximation, pattern recognition, and control systems. The core idea is to map low-dimensional linearly inseparable data into high-dimensional space, rendering them linearly separable. By carefully selecting the radial basis function and its parameters (such as the center point and width), RBFNN can effectively approximate complex nonlinear functions. As a neural network model, RBFNN possesses strong nonlinear fitting and generalization capabilities. Its unique structure and training algorithm give it significant advantages in dealing with complex nonlinear problems, which is why it has been widely applied in many fields [28,29].

##### Least Squares Support Vector Machine (LSSVM)

LSSVM is an extension of SVM proposed by Suykens et al. It employs the least squares method to find a hyperplane, mapping sample points to a high-dimensional space based on their feature vectors, thus enabling classification or regression predictions for the samples [30]. Compared to traditional SVM, LSSVM replaces the inequality constraints of the slack variable with equality constraints, transforming the problem into solving a system of linear equations, thereby simplifying the calculation process. LSSVM features fast convergence speed, minimal parameter adjustment, strong exploration ability, and high prediction accuracy, making it widely applied in finance, medicine, industry, meteorology, and other fields [31,32].

#### 2.3.5. Model Evaluation Method

The performance of the models was evaluated mainly by the coefficient of determination ($R^{2}$) and root mean square error (*RMSE*) [33,34]. The calculation formulas are as follows:

(7) $$R^{2}=\frac{\sum_{i=1}^{N}\left(x_{i}-\bar{x}\right)\left(y_{i}-\bar{y}\right)}{\sqrt{\sum_{i=1}^{N}{\left(x_{i}-\bar{x}\right)}^{2}+\sum_{i=1}^{N}{\left(y_{i}-\bar{y}\right)}^{2}}}$$

(8) $$RMSE=\sqrt{\frac{1}{N}\sum_{i=1}^{N}{\left(x_{i}-y_{i}\right)}^{2}}$$

where $x_{i}$ is the actual measured value, $y_{i}$ is the predicted value, $\bar{x}$ is the average measured value, and $\bar{y}$ represents the average predicted value, and N is the number of samples. $R^{2}$ is a value range of [0, 1]. The closer $R^{2}$ is to 1, and the smaller the *RMSE* is, the better the prediction performance of the regression model.

During the modeling process, if the values of $R_{C}^{2}$ and $R_{P}^{2}$ (determination coefficients of the calibration and validation sets) are large with a minimal difference, and the values of RMSEC and RMSEP (*RMSE* of the calibration and validation sets) are small and the difference is minimal, the model exhibits high reliability and credibility.

## 3. Results and Discussion

### 3.1. Sample Division

The samples were partitioned into a calibration set and a prediction set at a 3:1 ratio using the Sample Set Partition Based on Joint X-Y Distance (SPXY) algorithm. Table 1 displays the content statistics of each component in the dataset. The content range of each component within the calibration set encompasses the range present in the prediction set, suggesting that the division of the sample set is appropriate.

### 3.2. Spectral Extraction and Analysis

**Figure 3:**
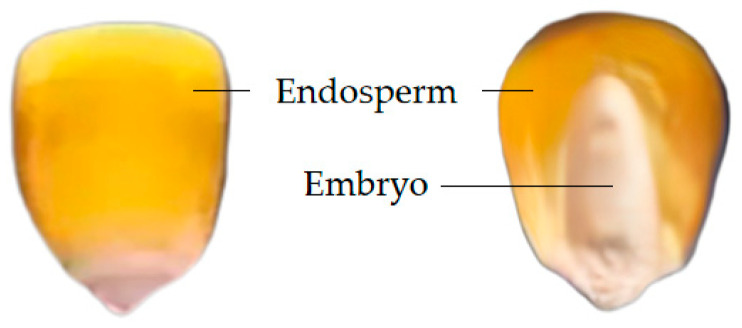
Structure of maize seed.

**Figure 4:**
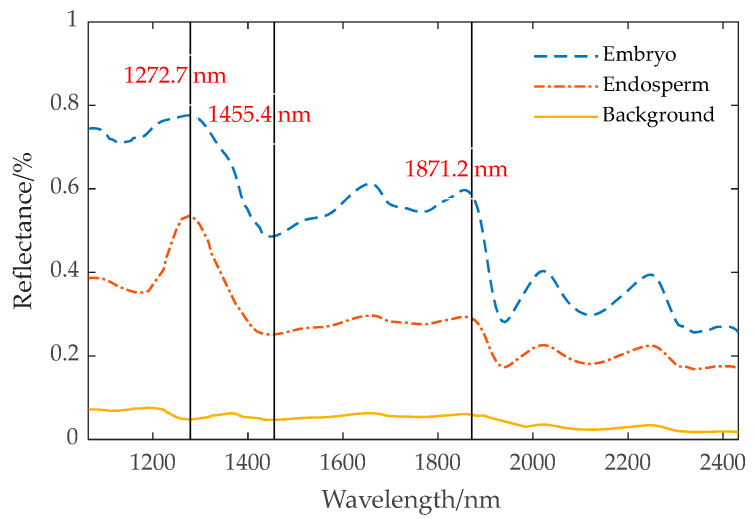
Average spectra of maize kernel embryo, endosperm, and background.

The surface structure of a corn kernel comprises the embryo and endosperm, as illustrated in Figure 3. In order to obtain the spectrum of the complete embryo structure region, the seed image is processed using threshold segmentation. Figure 4 shows the spectra of the embryo region, endosperm region, and background region of the corn kernel. 

**Figure 5:**
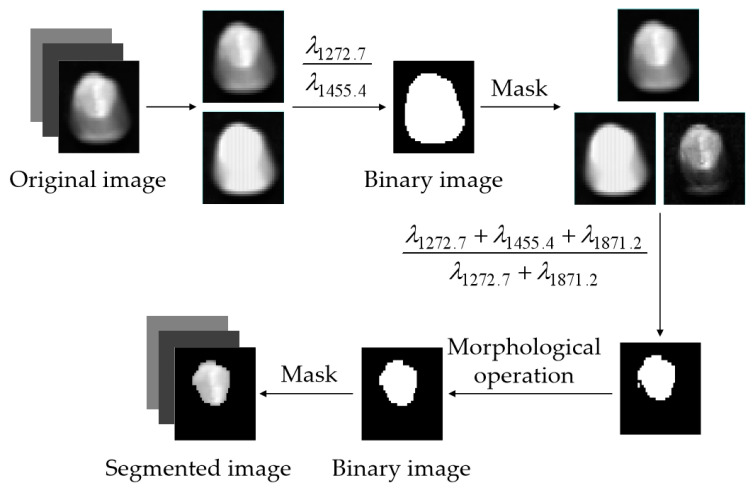
Flowchart of extracting the maize kernel embryo.

Figure 4 demonstrates that the spectral curve of the embryo exhibits a similarity to the trend observed in the endosperm, yet shows notable differences when compared to the background spectrum. At 1272.7 nm, 1455.4 nm, and 1871.2 nm, the reflectivity of the embryo and endosperm varies greatly, and the difference in reflectivity between 1272.7 nm and 1871.2 nm is smaller in the embryo structure area than in the endosperm structure area. It is difficult to distinguish the embryo and endosperm regions of some uneven-brightness samples by only using the reflection intensity of one band. The band ratio $\frac{\lambda_{1272.7}+\lambda_{1455.4}+\lambda_{1871.2}}{\lambda_{1272.7}-\lambda_{1871.2}}$ increases the band ratio of the embryo region and decreases the band ratio of the endosperm region. By threshold segmentation, the embryo structure can be effectively segmented from the image. Figure 5 shows the extraction process of the maize kernel embryo.

**Figure 6:**
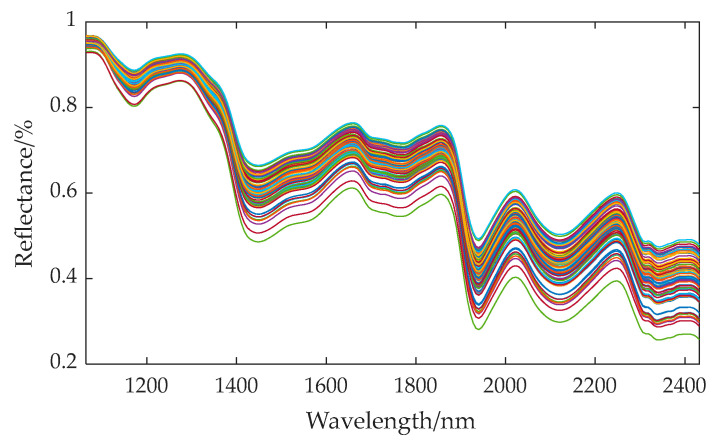
Average spectral curve of embryo structures of 80 varieties of corn seeds.

The wavelength range of hyperspectral data spans from 935.5 nm to 2539 nm, encompassing a total of 256 bands. Due to significant noise interference at the beginning and end of the spectral data during the acquisition process, these segments of data should be excluded from further analysis. Consequently, we utilized 218 bands, ranging from band 22 to band 239 (i.e., the spectrum spanning from 1065 nm to 2432 nm), for our analysis. The average spectral curve of the embryo structures of 80 varieties of corn seeds is shown in Figure 6.

Based on the spectral absorption curve, it is observed that the absorption peak located near 1450 nm corresponds to the first-order overtone (or fundamental) vibration of the NH bond present in proteins. Proteins are important components of corn seeds, which are composed of amino acids, and amino acid molecules contain NH bonds. Therefore, when these NH bonds vibrate at the fundamental frequency, an absorption peak near 1450 nm will be generated in the spectrum. The absorption peak near 1950 nm may be related to the carbohydrates in corn seeds, especially the second-order overtone vibration of C-O bonds. Carbohydrates are one of the main components of corn seeds, and they contain a large number of C-O bonds. Therefore, when these C-O bonds vibrate in the second-order overtone mode, corresponding absorption peaks will appear in the spectrum. The absorption peak near 2200 nm involves the combined vibration of OH and NH bonds [35]. In corn seeds, these two bonds are found in molecules such as water and proteins. When they vibrate in specific combinations, they may produce an absorption peak near 2200 nm on the spectrum. These absorption peaks provide us with useful information about the chemical composition and molecular structure of corn seeds. Therefore, it is feasible to quantitatively determine the main component content through the spectral curve of the seeds.

### 3.3. Spectral Preprocessing

The PLSR model takes into account the relationship between independent and dependent variables, allowing for regression modeling under conditions of severe multicollinearity among independent variables [36]. Therefore, the PLSR model was selected to compare the effects of different preprocessing methods. The regression model was evaluated using the fit index $R^{2}$ and the error index *RMSE*. With the original and preprocessed spectral data as inputs, four component content detection models were established, with the results shown in Table 2. In the table, $R_{C}^{2}$ and $R_{P}^{2}$ represent the determination coefficients for the training and test sets, respectively. The closer the value of $R^{2}$ is to 1, the better the model’s fit. Generally, a model with an $R^{2}$ above 0.4 is considered well-fitting. *RMSEC* and *RMSEP,* respectively, denote the root mean square errors of the training and testing sets, and the smaller they are, the superior the model’s fit.

**Figure 7:**
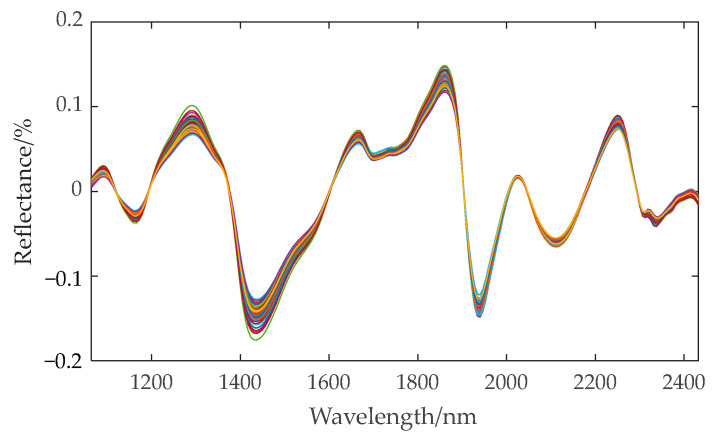
Spectral curve after DT preprocessing.

According to Table 2, on the whole, the prediction accuracy of the moisture content detection model is higher than that of the other three components. The prediction effect of fat content is relatively poor, with the minimum $R_{P}^{2}$ only having 0.6588. This is primarily because of the low fat content in corn kernels, which introduces certain errors in both physical and chemical measurements as well as hyperspectral measurements. The $R_{P}^{2}$ of the four component prediction sets for the original spectra without preprocessing are 0.9748, 0.7483, 0.8995, and 0.8249, respectively, which can better predict the content of each component. For different preprocessing methods, the detection results of different components vary inconsistently. After preprocessing with the MA and SG algorithms, the prediction performance of the model is inferior to that established using the original spectra. NOR improved the prediction effect of protein and starch content, but decreased the prediction effect of moisture and fat content. The BC, MSC, and SNV algorithms all improved the prediction accuracy for fat, protein, and starch content, but reduced the prediction accuracy for water content. Only when the DT algorithm is applied to preprocess the original spectra does the prediction performance of all components improve, with $R_{P}^{2}$ values being 0.9906, 0.8706, 0.9535, and 0.9145, respectively, and *RMSEP* values being 0.0393, 0.0606, 0.1057, and 0.2170, respectively. Therefore, in subsequent analysis, DT is selected for spectral preprocessing. The spectral curve after DT preprocessing is shown in Figure 7.

### 3.4. Feature Wavelength Extraction

**Figure 8:**
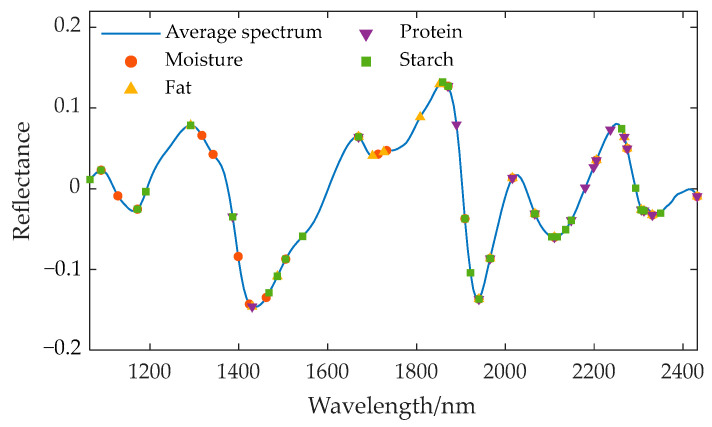
Location of the characteristic wavelengths extracted by SPA for different components.

**Figure 9:**
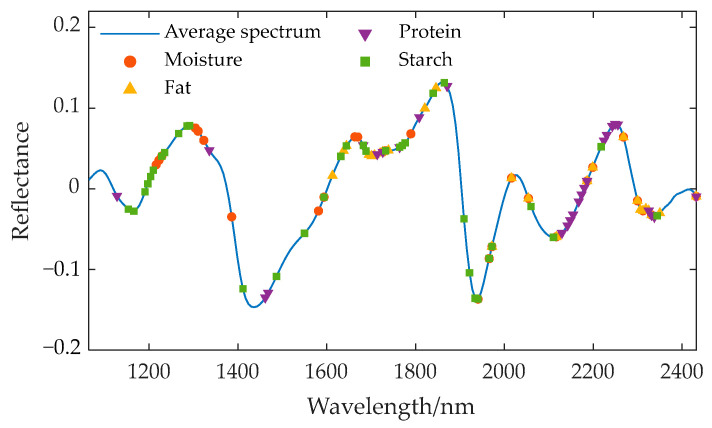
Location of the characteristic wavelengths extracted by UVE for different components.

**Figure 10:**
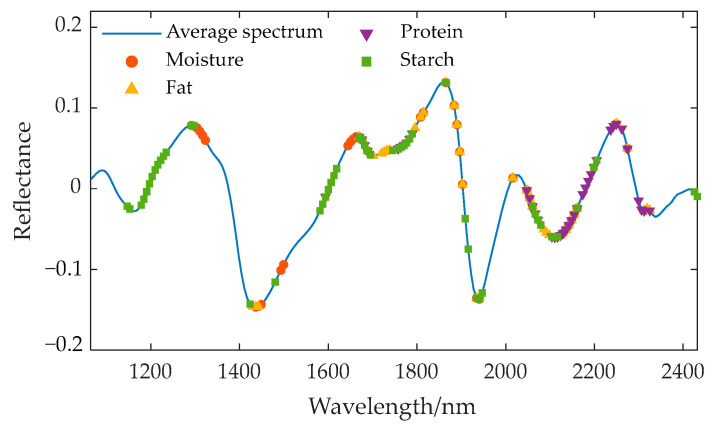
Location of the characteristic wavelengths extracted by CARS for different components.

Three feature wavelength selection algorithms—SPA, UVE, and CARS—were used to extract the characteristic wavelengths from the preprocessed spectra for various components. The wavelengths selected by SPA are associated with the identification of specific components, including moisture, fat, starch, and protein, as these wavelengths exhibit the greatest amount of information during projection, indicating their significant contribution to the prediction of target components. The wavelengths selected by UVE are those that make a significant contribution to the prediction of target components; that is, their regression coefficients are stable and significant, rather than representing random noise. The wavelengths selected by CARS are those that consistently demonstrate high significance across multiple rounds of sampling, and these bands make a stable and significant contribution to the prediction of target components. The characteristic wavelength positions corresponding to different components are displayed on the average spectral curve, and the extraction results are shown in Figure 8, Figure 9 and Figure 10.

In order to make quantitative predictions for four components simultaneously, the four sets of characteristic wavelengths extracted by each algorithm were combined into a single set. The SPA algorithm extracted a total of 51 characteristic wavelengths, the UVE algorithm extracted a total of 105 characteristic wavelengths, and the CARS algorithm extracted a total of 89 characteristic wavelengths. Table 3 presents the coefficients of determination for the prediction sets of the four components.

### 3.5. Establishment of a Model for Detecting the Content of Multiple Components in Maize Seeds

To ensure the generalizability and robustness of the model, this study selected machine learning models that stably perform on a relatively small dataset to detect the main component content of corn seeds. The PLSR algorithm has advantages in handling multivariate data, and can effectively extract the potential relationships between variables while reducing noise and redundant information in the data. BPNN and RBFNN, two neural network models, excel in nonlinear mapping and pattern recognition, and are capable of capturing complex relationships in data. LSSVM not only inherits the excellent generalizability of SVM in high-dimensional space, but also simplifies the calculation process through the least squares loss function, improving training efficiency. Therefore, this study utilized these four algorithms to construct models, with the aim of identifying the optimal model for detecting the compositional content in corn seeds.

The original spectra were preprocessed using the DT algorithm, followed by the extraction of characteristic wavelengths from the preprocessed spectral data. Subsequently, multi-output content detection models were established for PLSR, RBFNN, BPNN, and LSSVM, each based on both full-band spectra and characteristic wavelengths. The prediction performance of these models was evaluated using the coefficient of determination for the prediction set of each component. The results of the model predictions are presented in Table 4.

According to the model prediction results, it can be seen that the detection effect of water content in several components is the best, followed by starch content, and the prediction effect of fat content is relatively the worst. Using full-band spectra for modeling, the model prediction performance is better because the spectral data contains information about all seed components. Among the models established based on the full-band spectra, the PLSR model has the best prediction performance, with $R^{2}$ values of 0.9874, 0.9472, 0.9833, and 0.9532 for moisture, fat, protein, and starch content, respectively. Although the detection of fat content is not as good as that of the full-band RBFNN model, the detection of the other three components is higher than the RBFNN model.

Using feature selection modeling can significantly improve prediction accuracy and modeling efficiency, enhance model stability, and facilitate online real-time monitoring. However, it also has some disadvantages, such as a complex algorithm selection process, potential loss of useful information, sensitivity to initial conditions, and potentially high computational demands. Based on the prediction models of PLSR and RBFNN, from the perspective of prediction accuracy, the modeling performance of feature selection is not as good as that of full-spectrum modeling, indicating that during the feature selection process, some wavelengths with weak correlation to the target substance but containing useful information may be removed, resulting in a greater loss of spectral information related to the substance being tested. On the contrary, the BP neural network and LSSVM models have better prediction performance than the full-spectrum model when using feature selection for modeling. The best performance is achieved by the CARS-LSSVM model, with the $R^{2}$ values of the four components all improving compared to full-spectrum modeling, indicating that these two models can achieve more precise results with less sensitive information. It is not difficult to find that the prediction performance of the LSSVM model is relatively stable across several different input wavelengths, and the detection performance of the four components is relatively good.

### 3.6. Multi-Parameter Content Detection Model Based on PSO-LSSVM

The LSSVM model is easily influenced by the regularization parameter γ and the kernel function parameter μ during its usage. The magnitude of the regularization parameter γ influences the effectiveness of the SVM regression curve. If γ is small, underfitting is likely to occur, resulting in a flat regression curve and increased training error. Conversely, if γ is large, overfitting may ensue, leading to a decrease in training error but compromising the model’s generalization ability. The kernel function parameter μ represents the correlation degree of support vectors. When μ is small, the correlation among support vectors is low, making it prone to local optimization and resulting in overfitting of the SVM. Conversely, if μ is large, the connections between support vectors are tight, making it difficult to achieve the required accuracy in prediction, leading to underfitting of the SVM. Hence, optimizing these two parameters is crucial when establishing the LSSVM model. However, selecting these parameters based on experience increases computational load and the risk of falling into local extremes. Therefore, this study employs the Particle Swarm Optimization (PSO) algorithm to swiftly identify the optimal regularization parameter γ and kernel function parameter μ, thereby enhancing the efficiency of parameter tuning.

The steps for optimizing the hyperparameters of PSO are as follows: 

(1) Set the population size, iteration times, number of optimization parameters, and optimization range of PSO, and select the fitness function as the prediction accuracy of the training set; 

(2) Randomly initialize the velocity and position of each particle, and calculate the initial fitness value; 

(3) Calculate the fitness value of each particle to determine the global optimum and check whether the criteria are met. If the criteria are met, stop and output the relevant hyperparameters. If not, continue the iterative cycle; 

(4) For each particle, update its velocity and position, and limit the range of its position and velocity according to the boundary conditions; 

(5) Calculate the fitness value for each particle and update their respective historical optimal positions; 

(6) Update the global optimal position until the accuracy requirement is met, then stop the PSO algorithm and output the corresponding hyperparameter values.

The PSO-LSSVM algorithm was employed to develop a model for detecting the compositional content of corn seeds. The PSO parameters were set as follows: the learning factors, $c_{1}$ and $c_{2}$, were set to 1.5 and 1.7, respectively. The momentum coefficient, ω, was set to 1, the maximum number of evolutions was set to 200, and the particle swarm size was set to 20.

**Figure 11:**
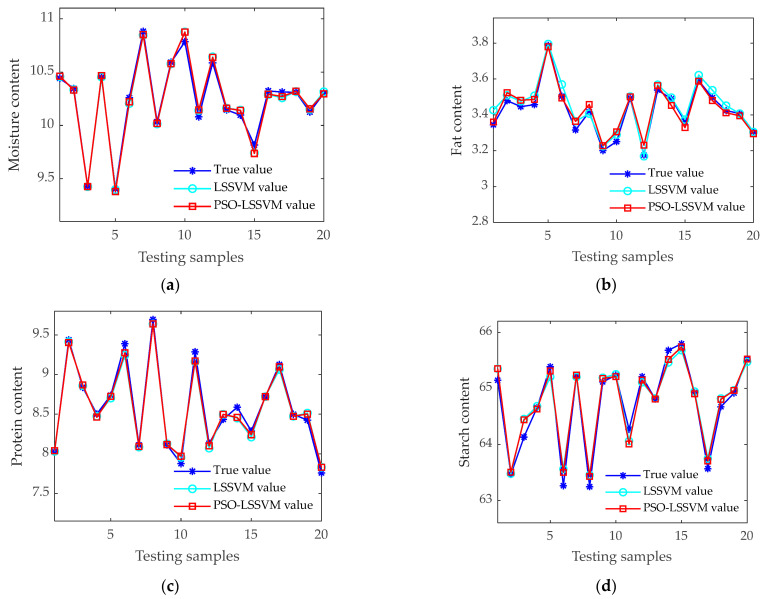
Comparison of test results between the LSSVM and PSO-LSSVM models. (**a**) Prediction results of moisture content model; (**b**) prediction results of fat content model; (**c**) prediction results of protein content model; (**d**) prediction results of starch content model.

A comparison was made between the prediction results of the LSSVM model and those of the PSO-LSSVM model, which utilizes optimization algorithms. The results of the two models are shown in Table 5, and the prediction results of the four components are shown in Figure 11.

According to Table 5, both models are able to make accurate predictions of the component content. PSO-LSSVM utilizes a parameter optimization algorithm to identify the optimal regularization parameter γ and kernel function parameter μ, thereby further enhancing the model’s prediction accuracy. The $R_{P}^{2}$ for the four components in the test set increased by 0.07%, 1.46%, 0.37%, and 0.95%, respectively. As can be seen from Figure 11, the fitting results of the PSO-LSSVM model are more accurate in reflecting the true values, resulting in higher accuracy in the detection of all four components. This demonstrates that the PSO-LSSVM method exhibits superior performance in the detection of corn seed component content, making it a suitable candidate as the optimal model for multi-parameter component detection in seed component content analysis.

## 4. Conclusions

This study utilized near-infrared hyperspectral imaging to determine the key quality indicators of 80 different varieties of corn seeds across a wide range of regions in China, including moisture, fat, protein, and starch content. Spectral reflectance data of the corn seeds were collected within the wavelength range of 935.5–2539 nm, and their physicochemical properties were accurately determined using chemometric analysis. A spectral-content dataset of corn seeds was constructed. Seven algorithms were used for spectral preprocessing, and the DT algorithm was found to be the optimal preprocessing algorithm for content detection. Three algorithms—SPA, UVE, and CARS—were used for feature extraction, and the PLSR, BP, RBF, and LSSVM models were established based on full-band and characteristic wavelength ranges to detect the content of the four components. The analysis results showed that the CARS-LSSVM algorithm had the best prediction performance. The PSO algorithm was used to further optimize the parameters of the LSSVM model, resulting in improved model prediction performance. The $R_{P}^{2}$ for the four components in the test set were 0.9884, 0.9490, 0.9864, and 0.9687, respectively. This indicates that hyperspectral technology combined with the DT-CARS-PSO-LSSVM algorithm can effectively detect the main component content of corn seeds.

To translate this research into practical use, we envision its integration into smart agriculture systems, enabling automated and intelligent seed quality evaluations that can enhance agricultural productivity, optimize planting decisions, and propel precision agriculture. With the ongoing development of portable or handheld HSI devices, field measurements may become feasible, facilitating more convenient seed quality assessments. However, challenges related to environmental light variations, device stability, and processing speed must be addressed. Additionally, farmer adoption and operational capabilities are crucial considerations for successful implementation. Future research should focus on collaborating with device manufacturers to develop field-ready HSI devices, conducting extensive field demonstrations, and enhancing farmer awareness and acceptance of these novel technologies.

In conclusion, this study not only establishes a robust scientific foundation for corn seed quality assessment but also opens new avenues for non-destructive testing technologies in agriculture and related fields. Ongoing efforts to optimize models and devices for wider real-world applications will contribute to the sustainable development of modern agriculture while acknowledging and addressing potential limitations and challenges.

## Figures and Tables

**Table 1:** Statistics of the content of each component in the calibration set and prediction set of corn seed samples.

Sample Set	Number of Samples	Parameter	Content/%			
			Moisture	Fat	Protein	Starch
Calibration set	60	Maximum	10.993	3.832	9.711	66.472
		Minimum	9.377	3.088	7.654	62.826
		Average	10.233	3.523	8.692	64.691
		Standard deviation	0.381	0.181	0.478	0.828
Validation set	20	Maximum	10.882	3.787	9.694	65.795
		Minimum	9.407	3.176	7.759	63.246
		Average	10.237	3.424	8.598	64.711
		Standard deviation	0.368	0.137	0.538	0.778

**Table 2:** Prediction results of PLSR models established based on different preprocessing methods.

Component	Pretreatment Method	PCs	Calibration Set		Validation Set	
			$R_{C}^{2}$	*RMSEC*	$R_{P}^{2}$	*RMSEP*
Moisture	No pretreatment	22	0.9928	0.0310	0.9748	0.0644
	MA	27	0.9893	0.0378	0.9611	0.0800
	SG	27	0.9925	0.0317	0.9733	0.0662
	NOR	13	0.9831	0.0475	0.8227	0.1708
	BC	25	0.9925	0.0317	0.8720	0.1451
	MSC	11	0.9925	0.0317	0.8708	0.1458
	SNV	11	0.9925	0.0317	0.8720	0.1451
	DT	22	0.9997	0.0061	0.9906	0.0393
Fat	No pretreatment	37	0.9052	0.0549	0.7483	0.0845
	MA	23	0.8757	0.0628	0.6588	0.0984
	SG	28	0.8993	0.0565	0.7309	0.0874
	NOR	20	0.9809	0.0246	0.6982	0.0925
	BC	26	0.9937	0.0141	0.8275	0.0699
	MSC	13	0.9937	0.0141	0.8305	0.0693
	SNV	13	0.9937	0.0141	0.8275	0.0699
	DT	26	0.9985	0.0068	0.8706	0.0606
Protein	No pretreatment	29	0.9563	0.1032	0.8995	0.1554
	MA	36	0.9286	0.1320	0.8684	0.1778
	SG	40	0.9532	0.1069	0.8964	0.1578
	NOR	16	0.9933	0.0404	0.9265	0.1329
	BC	36	0.9981	0.0214	0.9365	0.1235
	MSC	13	0.9981	0.0214	0.9361	0.1239
	SNV	13	0.9981	0.0214	0.9365	0.1235
	DT	27	0.9994	0.0117	0.9535	0.1057
Starch	No pretreatment	24	0.9162	0.2421	0.8249	0.3106
	MA	29	0.8744	0.2964	0.7372	0.3806
	SG	19	0.9094	0.2517	0.8226	0.3127
	NOR	23	0.9942	0.0635	0.8793	0.2579
	BC	27	0.9980	0.0372	0.8986	0.2364
	MSC	20	0.9980	0.0373	0.8982	0.2369
	SNV	19	0.9980	0.0372	0.8986	0.2364
	DT	12	0.9979	0.0382	0.9145	0.2170

**Table 3:** The number of wavelengths selected based on different feature wavelength extraction algorithms.

Algorithm	Moisture	Fat	Protein	Starch	Total
None	—	—	—	—	218
SPA	24	14	10	24	51
UVE	27	14	25	35	105
CARS	41	15	39	53	89

**Table 4:** Prediction results of various components in different models.

Model	Feature Wavelength Extraction Algorithm	$R_{P}^{2}$			
		Moisture	Fat	Protein	Starch
PLSR	None	0.9874	0.9472	0.9833	0.9532
	SPA	0.9876	0.8017	0.9587	0.8692
	UVE	0.9900	0.8855	0.9792	0.9671
	CARS	0.9914	0.8215	0.9615	0.9395
BPNN	None	0.9527	0.7398	0.9250	0.8906
	SPA	0.8952	0.7994	0.9121	0.8941
	UVE	0.9522	0.9212	0.9701	0.8753
	CARS	0.9628	0.8680	0.9594	0.8621
RBFNN	None	0.9847	0.9707	0.9696	0.8697
	SPA	0.9804	0.7335	0.7495	0.9479
	UVE	0.9502	0.7903	0.9460	0.8387
	CARS	0.9744	0.9192	0.9346	0.9499
LSSVM	None	0.9822	0.8799	0.9775	0.9453
	SPA	0.9841	0.8696	0.9715	0.9520
	UVE	0.9857	0.9097	0.9768	0.9619
	CARS	0.9877	0.9344	0.9827	0.9592

**Table 5:** Results of LSSVM and PSO-LSSVM models.

Model	Component	Calibration Set		Validation Set	
		$R_{C}^{2}$	*RMSEC*	$R_{P}^{2}$	*RMSEP*
LSSVM	Moisture	0.9981	0.0167	0.9877	0.0408
	Fat	0.9922	0.0317	0.9344	0.0351
	Protein	0.9938	0.0377	0.9827	0.0707
	Starch	0.9874	0.0931	0.9592	0.1570
PSO-LSSVM	Moisture	0.9986	0.0140	0.9884	0.0396
	Fat	0.9925	0.0301	0.9490	0.0309
	Protein	0.9960	0.0303	0.9864	0.0627
	Starch	0.9908	0.0796	0.9687	0.1376

## Data Availability

All relevant data presented in the article are stored according to institutional requirements and, as such, are not available online. However, all data used in this manuscript can be made available upon request to the authors.
